# Research on Micro-Vibration Analysis of Segmented Telescope Based on Opto-Mechanical Integration

**DOI:** 10.3390/s25061901

**Published:** 2025-03-19

**Authors:** Kangmin Wen, Lingjie Wang, Xuefeng Zeng, Yang Liu, Wenyan Li, Lianqiang Wang, Wei Sha, Di Zhou

**Affiliations:** 1 Changchun Institute of Optics, Fine Mechanics and Physics, Chinese Academy of Sciences, Changchun 130033, China; wenkangmin0920@163.com (K.W.); zxf@ciomp.ac.cn (X.Z.); liuyang_08_22@163.com (Y.L.); lwy_978@163.com (W.L.); wanglianqiang@ciomp.ac.cn (L.W.); shawei@ciomp.ac.cn (W.S.); 15504303690@163.com (D.Z.); 2 University of Chinese Academy of Sciences, Beijing 100049, China; 3 State Key Laboratory of Advanced Manufacturing for Optical Systems, Changchun 130033, China

**Keywords:** segmented telescope, micro-vibration, opto-mechanical integration analysis, wavefront error

## Abstract

Aiming at the inherent nature and complexity of the influence of in-orbit micro-vibration in the imaging quality of segmented telescopes, a dynamic full-link opto-mechanical integration analysis method is proposed. The method is based on the measured micro-vibration signals of the infrared refrigerator, using the finite element method to perform the transient response analysis of the opto-mechanical system in Patran/Nastran software. The interface tool is written by Matlab to achieve the calculation of rigid body displacement and real-time data interaction with Zemax. The results show that when the working wavelength is 1 μm, the optical system has a wavefront error Root-Mean-Square value of less than 0.071λ in 4 s. Evaluating the effect of micro-vibration on the imaging quality of the system in terms of the peak ratio of the point spread function. When the exposure time was 2 s, the ratio maximum values of 0.4628 and 0.6207 were reached for the X-axis and Y-axis, respectively. The method provides an important reference basis for the evaluation of imaging quality of an optical system under micro-vibration environment with a long exposure time.

## 1. Introduction

With the increasingly high requirements for the resolution and imaging quality of space telescopes, space telescopes are developing in the direction of large aperture, long focal length and large field of view. According to the Rayleigh criterion, the improvement of telescope resolution can be realized by increasing the aperture of the primary mirror, but the large-size primary mirror will bring problems such as processing and manufacturing difficulties and excessive transportation cost, etc. To solve the above problems, large-aperture segmented telescopes [1,2,3] came into being, and ground-based telescopes such as KECK I/II [4], GTC [5], GMT [6], and TMT [7], space-based telescope JWST [8,9] and other telescopes have adopted the design method of segmented primary mirror.

However, for segmented telescopes, only the co-focusing and co-phasing between the sub-mirrors can ensure that the segmented mirror achieves the same imaging quality as its single mirror of the same aperture [10,11,12,13].Vievard [14] proposed the ELASTICS algorithm to be able to estimate the pupil-plane phase maps with an RMS accuracy of λ/20, and a linearized phase diversity algorithm LAPD for the fine-phase modes, with the final ELASTICS state is used as the starting point for fine phasing with LAPD. The on-orbit deployment of JWST makes the JWST co-phase wavefront detection and control system more complex. The final JWST primary mirror integrated surface shape accuracy is 23.2 nm, the sub-mirror Tip & Tilt error correction accuracy can be better than 0.028″, and the Piston error correction accuracy has reached tens of nanometers [15,16,17].

When the satellite is in the in-orbit working state, it will be affected by the fluctuation of the space environment and the perturbation of the internal vibration source of the satellite and generate micro-vibration [18,19,20]. Aiming at the source of micro-vibration, Masterson et al. [21] used the theoretical derivation to represent the structural elasticity and damping of the system by a linear spring and a linear damper, respectively, and considered that the flywheel disturbance mainly originated from the rotor’s dynamic imbalance and static imbalance, so as to establish an analytical model. Le [22] summarized the main reasons for the flywheel disturbance, and carried out the ground disturbance test of the flywheel and the in-orbit test, and established an analytical model based on the statistical analysis of the test data. orbit test, and established the flywheel disturbance model based on the statistical analysis of the test data. Luo [23] analyzed and studied the broadband vibration output during flywheel operation by using quantile statistics to establish the grading standard of flywheel micro-vibration, which can provide a theoretical and engineering basis for the development and screening of super-static flywheel. Toyoshima [24] conducted space measurements of the angular micro-vibration of platform jitter in the ground-star laser communication link of the Optical Communication Engineering Test Satellite (OCETS) and compared the results with those of ground tests. The measurement results provide a reference for the estimation of angular jitter and the design of tracking control loops for space laser communication systems.

Micro-vibration is characterised by small energy, broad spectrum, inherent nature and difficult to suppress. It will cause the rigid body displacement and the surface shape change of the optical elements within the optical system, thus directly affecting the imaging quality. Among them, the rigid body displacement of optical elements has the most significant impact on image quality. However, for the segmented primary mirror optical structure, micro-vibration leads to more complex changes in the positional relationship between the sub-mirrors. Yaitskova [25] analyzes the point spread function of a segmented mirror affected by random tilt errors. In addition to the evaluation of the Strehl ratio, key features such as the intensity and position of scattering spots and secondary peaks, as well as the relative energy distribution between these features, were considered. Chen [26] derived the relationship between the wavefront variance and the number of sub-apertures in the presence of various aberrations in a single sub-aperture of an optical synthetic aperture imaging system. The results show that piston error has the greatest impact on the system, followed by defocus error, tilt error, spherical aberration, coma aberration and image dispersion.

Since it is difficult to carry out on-orbit testing of space telescope systems, it is generally necessary to carry out simulation analysis and ground testing first. In the 1980s, Honeywell proposed the opto-mechanical integration analysis method, in which an important link is the data transmission between finite element analysis software and optical design software. Many international scholars have carried out research on opto-mechanical integration integration analysis and achieved corresponding results. For example, the Massachusetts Institute of Technology (MIT) developed the opto-mechanical integration analysis software DOCS based on MATLAB R2016b, and used it to perform integration analysis of NEXUS [27,28], and applied DOCS to the micro-vibration integration analysis of NGST and other very large space telescopes, etc. NASA has also developed the opto-mechanical integration analysis software IME to perform opto-mechanical integration analysis of JWST [29], which can analyze the imaging quality under the effect of jitter and temperature loading. Scholar Shi [30] used Zernike polynomials as the interface to write an optical-machine interface program for data interaction, and analyzed the optical performance of the primary mirror of a telescope under the effect of microturbulence. Liu [31] used opto-mechanical integration analysis to conduct micro-vibration studies on a large aperture long focal length optical space remote sensing cam era, and realized a comprehensive evaluation of the imaging quality of the optical system under the action of micro-vibration of the flywheel.

Large aperture segmented telescopes have high-resolution imaging capabilities, however, in the extreme complexity of the space environment, they are susceptible to deterioration of imaging due to micro-vibrations during operation. Especially in long exposure missions, this can lead to increased wavefront errors, image blurring and resolution degradation. To ensure high resolution and reliability, the effects of micro-vibration must be considered at the design stage and opto-mechanical integration analysis must be performed. Although the analyses for optical systems where the primary mirror is a whole mirror are more mature, the precision integration analyses of segmented primary mirror optical systems still face challenges. There is also an urgent need to address the dynamic performance analysis in long exposure modes. These studies are not only crucial for the development of large-scale optoelectronic equipment, but also can provide leadership and reference for the environmental adaptability studies of other space precision equipment. Therefore, the study of the effect of micro-vibration on the imaging quality of segmented telescopes is of great theoretical and practical significance, and is an urgent problem that needs to be solved at present.

Based on the above, this paper takes the segmented telescope as the research object, theoretically analyzes the influence of the positional error of a single sub-mirror on the wavefront error (WFE) and carries out simulation verification. At the same time, we employ opto-mechanical integration analysis to simulate and analyze the entire link structure of the optical system under micro-vibration conditions. The degradation of imaging quality in the segmented telescope caused by micro-vibration is evaluated using the point spread function (PSF) as the evaluation index. This analysis is conducted at different exposure times, providing valuable references for assessing image quality under long exposure conditions.

The rest of the paper is organized as follows. Section 2 introduces the imaging theory of segmented telescope. The simulation verification of the effect of the positional error of the singlet mirror on the WFE is performed in Section 3. Section 4 presents the opto-mechanical integration analysis of the complete segmented telescope and Section 5 discusses the results. Section 6 concludes the paper and summarizes the subsequent work.

## 2. Segmented Telescope Imaging Theory

### 2.1. Segmented Mirror Imaging Optical Model

**Figure 1:**
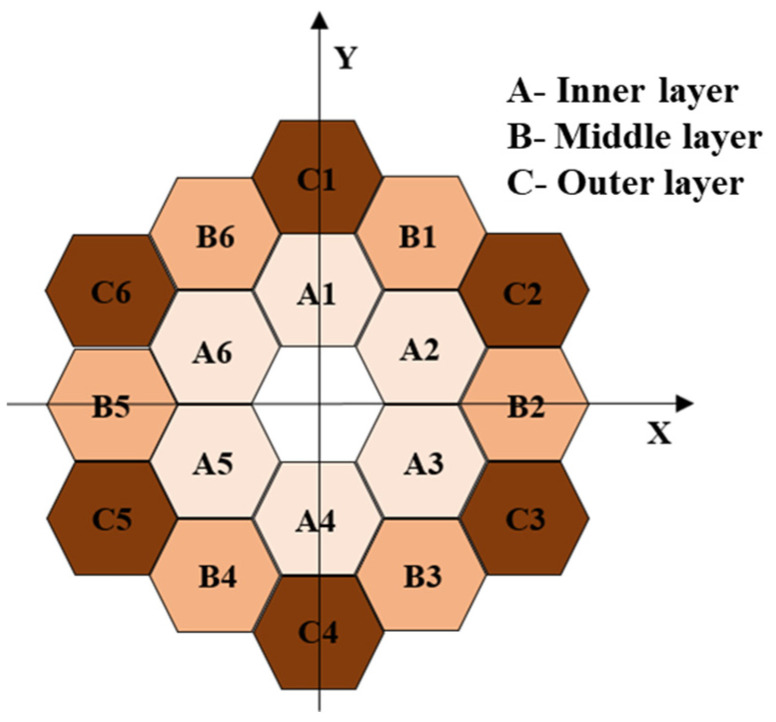
Schematic diagram of the structure of the segmented primary mirror.

The segmented telescope primary mirror consists of 18 hexagonal sub-mirrors segmented together in the arrangement shown in Figure 1. When the incident beam is parallel light of unit amplitude, the aperture function of the segmented primary mirror can be expressed as:

(1) $$f\left(x,y\right)=\sum_{j=1}^{N}f_{j}\left(x-x_{j},y-y_{j}\right)=\sum_{j=1}^{N}a_{j}\left(x-x_{j},y-y_{j}\right)\cdot \exp\left(i\phi_{j}\left(x-x_{j},y-y_{j}\right)\right)$$

where $\left(x_{j},y_{j}\right)$ is the center coordinate of the jth sub-mirror, $f_{j}\left(x-x_{j},y-y_{j}\right)$ is the aperture function of the jth sub-mirror, and $\phi_{j}\left(x-x_{j},y-y_{j}\right)$ is the phase function of the jth sub-mirror. When the sub-mirror has position error or face shape error, the phase $\phi_{j}$ of the corresponding sub-mirror will be changed accordingly. $a_{j}\left(x-x_{j},y-y_{j}\right)$ is the transmission intensity function, expressed as the following equation:

(2) $$a_{j}\left(x-x_{j},y-y_{j}\right)=\left\{\begin{array}{c} 1,\;\mathrm{inside}\\ 0,\;\mathrm{outside} \end{array}\right.$$

The complex amplitude distribution in the image plane can be expressed as the Fourier transform of the aperture function, viz:

(3) $$U\left(x_{f},y_{f}\right)=\frac{1}{\lambda f}\iint \sum_{j=1}^{N}f_{j}\left(x-x_{j},y-y_{j}\right)\cdot \exp\left[i2\pi \left(xf_{x}+yf_{y}\right)\right]\mathrm{dxdy}$$

where λ is the wavelength, f is the focal length, $\left(x_{f},y_{f}\right)$ is the image plane coordinates, $\left(f_{x},f_{y}\right)$ is the frequency domain coordinates, and the PSF of the system is the mode square of the complex amplitude.

When the sub-mirrors are all in the ideal position, the phase error of the sub-mirrors $f\left(x-x_{j},y-y_{j}\right)=0$, the PSF of a segmented mirror can be represented as the product of two factors:

(4) $$F_{\mathrm{PSF}}(x_{f},y_{f})={\left(\frac{\mathrm{AN}}{\lambda \cdot f}\right)}^{2}G\left(x_{f},y_{f}\right)F_{\mathrm{s-PSF}}(x_{f},y_{f})$$

where A is the area of the monolithic sub-mirror. $G\left(x_{f},y_{f}\right)$ the Fourier transform of the segmentation grid, usually a periodic function of sharp peaks, each of which is the Fourier transform of the full telescope aperture. $F_{\mathrm{s-PSF}}(x_{f},y_{f})$ is the PSF of the monolithic sub-mirror.

### 2.2. Derivation of WFE from Sub-Mirror Position Error

**Figure 2:**
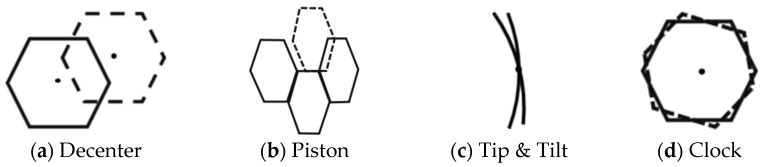
Schematic diagram of sub-mirror splicing error.

The positional errors induced by the six degrees of freedom corresponding to each sub-mirror cause the corresponding phase function $\phi_{j}$ to change. Setting the system optical pupil surface in the primary mirror, various positional errors will introduce WFE in the optical pupil surface, such as Figure 2 shows four kinds of positional errors, and the phase function $\phi_{j}$ generated by various positional errors is analyzed below.

#### 2.2.1. Influence of X, Y Decenter Error on Phase $\phi_{j}$

If the ideal coordinates of a point on the sub-mirror are $\left(x_{0},y_{0},z_{0}\right)$, and the coordinates after the decenter transformation are $\left({x_{0}}^{\prime },{y_{0}}^{\prime },{z_{0}}^{\prime }\right)$, its decenter along the X and Y axis dx, dy and the coordinates satisfy the following relationship:

(5) $$\begin{array}{l} {x_{0}}^{\prime }=x_{0}+\mathrm{dx}\\ {y_{0}}^{\prime }=y_{0}+\mathrm{dy} \end{array}$$

The WFE caused by the sub-mirror decenter is the difference between the vector heights Sag before and after the change, and in the practical case dx, dy are small, $dx^{2}\ll x_{0}\mathrm{dx}$, $dy^{2}\ll y_{0}\mathrm{dy}$, can be simplified as:

(6) $$\phi_{\mathrm{j-Decenter}}=\frac{2\pi }{\lambda }\left({z_{0}}^{\prime }-z_{0}\right)\approx \frac{2\pi }{\lambda }\left(\frac{\mathrm{dx}}{r_{0}}x_{0}+\frac{\mathrm{dy}}{r_{0}}y_{0}\right)$$

The decenter error of the sub-mirrors can be equated to the tilt error, and the tilt factor is the ratio of the decenter to the radius of curvature of the vertex, but because of the $\mathrm{dx},\;\mathrm{dy}\ll r_{0}$, the decenter error does not have a significant effect on the WFE of the system.

#### 2.2.2. Influence of Z Piston Error on Phase $\phi_{j}$

When the offset of a sub-mirror along the optical axis direction is dz, the Piston error value is twice of the mutual misalignment value between the sub-mirrors along the optical axis direction 2dz, resulting in a WFE that can be expressed as:

(7) $$\phi_{\mathrm{j-Piston}}=\frac{2\pi }{\lambda }\cdot 2d_{z}=\frac{4\pi }{\lambda }d_{z}$$

The WFE is linearly related to the Piston error and manifests itself as a translation of the entire wavefront.

#### 2.2.3. Influence of Tip & Tilt Error Around X and Y Axis on Phase $\phi_{j}$

The X and Y axis of the sub-mirror intersect at the center point of the sub-mirror, $\left(x_{j},y_{j}\right)$, and the center point of the sub-mirror remains unchanged when the tilt error occurs. When the sub-mirror is tilted around the X-axis α, around the Y-axis β, the WFE caused by the tilt of the sub-mirror can be expressed as if only the first-order term is retained:

(8) $$\phi_{\mathrm{j-Tip}\&\mathrm{Tilt}}=\frac{2\pi }{\lambda }\left[\left(y-y_{j}\right)\sin\alpha +\left(x-x_{j}\right)\sin\beta \right]$$

From the above equation, it can be seen that the WFE formed by tilting around the X and Y axis are independent of each other, and this error is directly reflected in the wavefront tilt.

#### 2.2.4. Influence of Clock Error Around the Z-Axis on Phase $\phi_{j}$

If the sub-mirror rotates γ around the Z-axis with the center point $\left(x_{j},y_{j}\right)$ as the origin, when γ is very small, only the first-order term is retained, and the WFE due to the Clock error can be obtained as:

(9) $$\phi_{\mathrm{j-Clock}}=\frac{2\pi }{\lambda }\left[-\frac{y_{j}}{r_{0}}x_{0}+\frac{x_{j}}{r_{0}}y_{0}\right]\sin\gamma$$

From the above equation, it can be concluded that the Clock error of the sub-mirror is related to the angle of rotation of the sub-mirror and the position in which the sub-mirror is located.

### 2.3. Theoretical Analysis of the Effect of Micro-Vibration on Image Quality Under Exposure

Ideally, the width of the PSF is mainly determined by the diffraction limit, and for a circular aperture, the full-width-half-height (FWHM) of the PSF is approximately 1.22λ/D, where λ is the wavelength of the light wave and *D* is the diameter of the aperture. Under micro-vibration conditions, the width and height of a point PSF can be significantly affected. The vibration causes the optical axis to shift, causing the width of the PSF to increase. At the same time, the intensity of the central peak of the PSF decreases due to the dispersion of light energy over a wider area, in addition to increasing the intensity of the secondary peaks.

Assuming that the point spread function of the optical system for ideal imaging is $h\left(x,y\right)$, and In the integral time, the image shift in the x direction generated by micro-vibration is $\varepsilon_{x}\left(t\right)$, and the image shift in the y direction is $\varepsilon_{y}\left(t\right)$, the energy distribution of the diffuse spot of the image point at the moment of t is:

(10) $$g\left(x,y,t\right)=h\left(x,y\right)*\delta \left(x-\varepsilon_{x}\left(t\right),y-\varepsilon_{y}\left(t\right)\right)$$

where is the $\delta \left(x,y\right)$ Dirac symbol and ∗ is the convolution symbol. After the introduction of the image shift, the energy of the diffuse spot is normalized to obtain the point spread function at long exposure time as follows:

(11) $$\mathrm{LSF}\left(x,y\right)=\frac{1}{t_{1}}\int_{t_{0}}^{t_{0}+t_{1}}g\left(x,y,t\right)\;\mathrm{dt}=h\left(x,y\right)*\frac{1}{t_{1}}\int_{t_{0}}^{t_{0}+t_{1}}\delta \left(x-\varepsilon_{x}\left(t\right),y-\varepsilon_{y}\left(t\right)\right)\mathrm{dt}$$

where $t_{0}$ is the initial integration moment and $t_{1}$ is the integration time. During the imaging process, when micro-vibration acts on the optical system, the center position of the dot diffusion function will deviate from the ideal position. As a result, the micro-vibration will cause the diffuse spot diameter of the image point to become larger and the center energy to decrease, making the image blur geometrically larger.

## 3. Effect of Sub-Mirror Position Error on WFE

**Figure 3:**
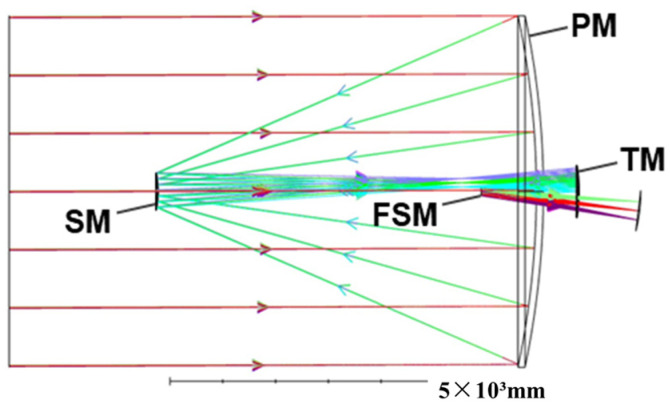
Layout of segmented telescopes.

The optical model is established for simulation and analysis according to the optical design of the James Webb telescope, which is a coaxial triple-reflector achromatic telescope with the primary mirror split as shown in Figure 1 (hexagonal split), and the layout of the optical system as shown in Figure 3. The different colored arrows in the figure correspond to different fields of view of the light. Among them, the primary mirror (PM), secondary mirror (SM), and tertiary mirror (TM) are designed using the quadratic surface reflector design method, and in order to make the structure of the optical system more compact and reasonable, the fine steering mirror (FSM) is utilized for the folding imaging of the optical path.

The specific structural parameters of the optical system are shown in Table 1.

Compared to non-segmented primary mirror optical systems with the same structure, segmented primary mirrors with misalignments cannot achieve the effective aperture size of a monolithic mirror due to the presence of gaps between segments. This causes the MTF of the optical system to decrease at mid-frequency positions, but the segmented mirror increases the cutoff frequency of the entire system. Where, for this optical system, the operating wavelength of the system is 1 μm. When the wavefront difference RMS of the optical system is less than 1/14λ, the image quality is considered to be good, and the diffraction limit can be considered to have been approached. Based on this method, the influence of different position errors of sub-mirror on wavefront of optical system is simulated.

### 3.1. Decenter in X and Y Direction

**Figure 4:**
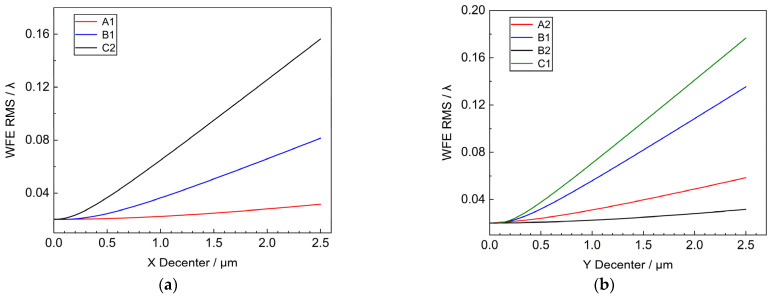
Systematic WFE RMS versus sub-mirror Decenter:(**a**) WFE RMS vs. X Decenter; (**b**) WFE RMS vs. Y Decenter.

The sub-mirrors at different positions were selected so that they were eccentrically shifted along the X and Y directions radially within the range of 2.5 μm, and the variation of the systematic WFE RMS with respect to the amount of decenter was obtained as shown in Figure 4.

For the sub-mirror X-direction decenter, three sub-mirrors with different X-positions, A1, B1, and C2, are selected respectively, and when the sub-mirror X-direction decenter error is 2.5 μm, the RMS of the system’s WFE is 0.0316λ, 0.0815λ, and 0.1564λ, respectively. Among them, the X-coordinate value of C2 is the largest, and the theoretical and simulation results indicate that the sub-mirror X-direction decenter error has the largest effect on the system’s WFE RMS of the system WFE RMS.

For the sub-mirror Y-direction decenter, similar to the X-direction decenter, four sub-mirrors with different Y-positions, A2, B1, B2, and C1, are selected respectively, and when the sub-mirror Y-direction decenter error is 2.5 μm, the RMS of the system’s WFE is 0.0317λ, 0.0585λ, 0.1354λ, 0.1766λ, respectively, where the C1 has the largest influence on the RMS of the system’s WFE.

### 3.2. Piston in Z Direction

**Figure 5:**
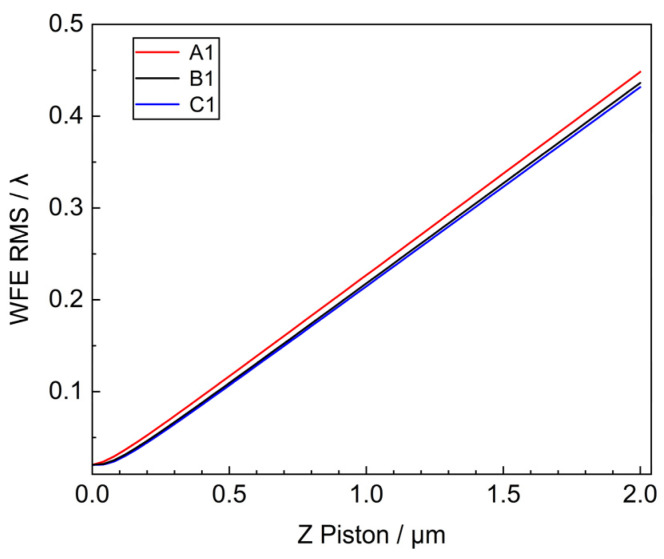
System WFE RMS vs. sub-mirror Piston.

The outer, middle, and inner circle mirrors were selected to be offset along the Z-axis direction in the range of 1 μm, respectively. Their systematic WFE RMS variations are shown in Figure 5.

For the sub-mirror piston along the Z-direction, the WFE RMS is approximately linear with the Piston error. When the Piston error of A1, B1, and C1 is 2 μm, the RMS values are 0.4482λ, 0.4362λ, and 0.4317λ, respectively.

### 3.3. Tip & Tilt Around the X and Y Axis

In order to visually compare and analyze the influence of different positions of sub-mirror Tip & Tilt on the WFE, a local coordinate system was established with the center of each sub-mirror as the origin, with the Z’-axis as the normal direction of the center of the sub-mirror, and the X’ and Y’-axis located in the tangent plane of the center of the sub-mirror and perpendicular to each other, respectively.

**Figure 6:**
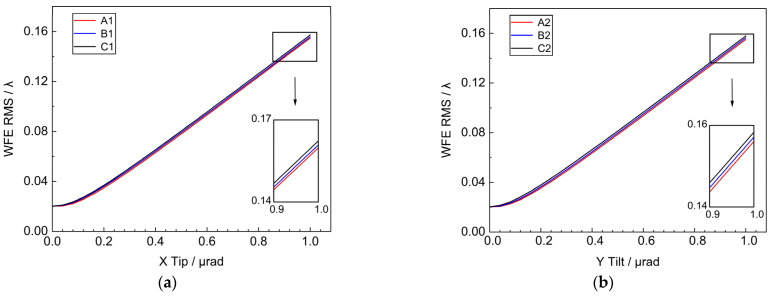
Systematic WFE RMS versus sub-mirror Tip & Tilt (**a**) WFE RMS vs. Tip; (**b**) WFE RMS vs. Tilt.

Taking the center point of the sub-mirror as the origin of the local coordinate system tip & tilt around the X’ and Y’ axis. The sub-mirror tipping(tilting) ranges are all within 1 μrad, and three different positions of the sub-mirror tip (tilt) are taken respectively to get the variation of the RMS of the system WFE as shown in Figure 6. It can be seen that within 1 μrad, the RMS of the WFE is approximately linearly related to the tip (tilt) angle of the sub-mirror. When the sub-mirror tilt is 1 μrad, the RMS is 0.157λ. The tilt of the sub-mirror causes misalignment between the sub-mirrors, directly disrupting the co-phasing and co-focusing conditions of the primary mirror. This error needs to be strictly controlled to ensure good imaging quality.

### 3.4. Clock Around the Z-Axis

**Figure 7:**
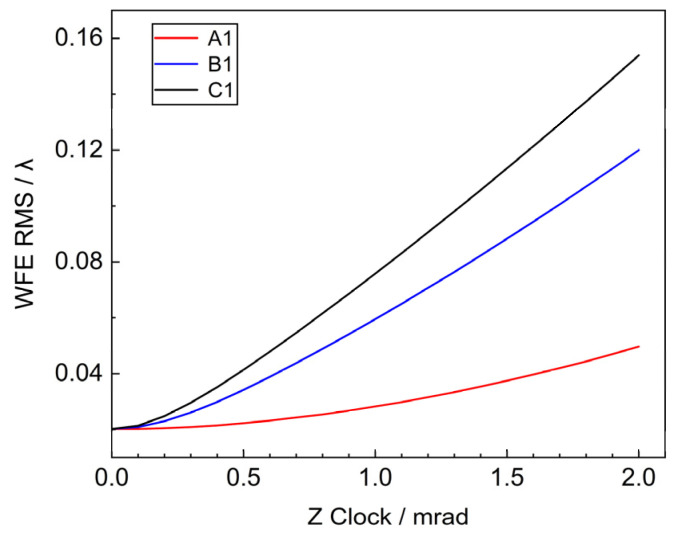
System WFE RMS vs. sub-mirror Clock.

The Z’ axis passing through the origin of the local coordinate system of each sub-mirror is taken as the rotation axis, and the system’s WFE RMS changes are obtained by rotating within 2 mrad, as shown in Figure 7.

The outer, middle and inner circle mirrors are selected, and when the rotational error of Z’ of the sub-mirror is 2 mrad, the corresponding WFE RMS are 0.1539λ, 0.12λ, 0.0497λ, respectively, which are consistent with the theory. When the rotation angle is fixed, the influence of Clock on the system WFE increases with the distance of the sub-mirror from the center of the primary mirror. Additionally, the error tolerance for rotation around the Z’ axis is 103 times greater than that for tilting around the X’ and Y’ axes. So more attention should be paid to the Tip & Tilt error during the mounting and tuning process.

## 4. Opto-Mechanical Integration Analysis

**Figure 8:**
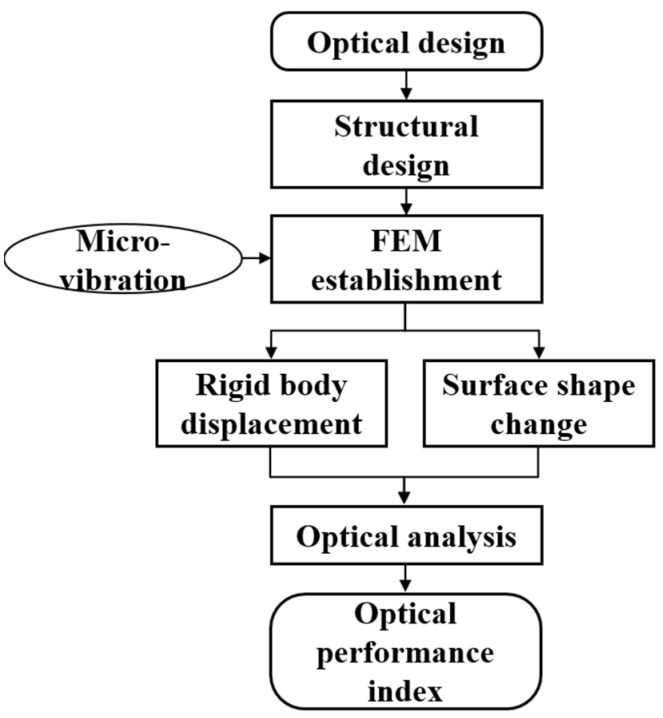
Flowchart of opto-mechanical integration analysis.

ZEMAX, as optical analysis software, cannot independently evaluate the image quality of an optical system under external loads. Therefore, multiple software tools are required to jointly transfer data. This collaboration is necessary to complete the full process of opto-mechanical integration analysis. Figure 8 shows the flow chart of integrated optical and mechanical analysis. Firstly, NX software is used to establish the mechanical model of the segmented telescope according to its optical model. Nastran software is used to conduct the structural finite element analysis before processing and Patran is used to conduct the post-processing analysis. The node position change obtained by finite element analysis is calculated as displacement data by using the optical machine interface programmed in MATLAB. Finally, the optical performance index of the system at various moments are obtained.

### 4.1. Micro-Vibration Source

**Figure 9:**
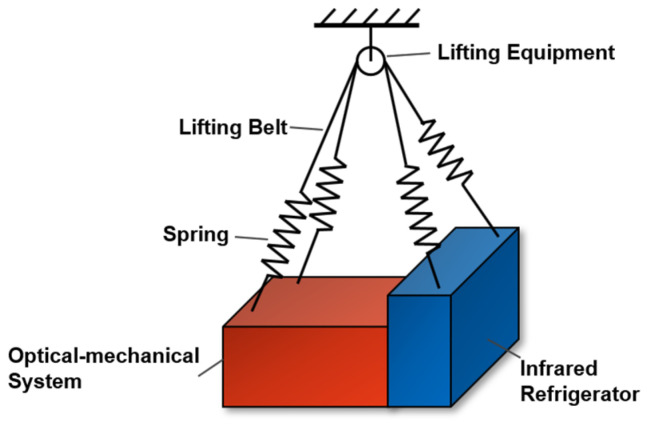
Micro-vibration signal test platform.

The size of the optical system is large, and in orbit it is susceptible to micro-vibration from the space environment and the internal micro-vibration sources of the system. Among them, solar radiation pressure noise and gravity field changes are small and negligible, and the thermal deformation period is the same as the orbital period. At the same time, the infrared refrigerator on the satellite has been running for a long time to produce micro-vibrations. As shown in Figure 9 the optical-mechanical system is fixed with the infrared refrigerator and suspended in the suspension system. A low stiffness suspension system composed of lifting belts and springs was used to simulate the zero gravity environment in orbit.

**Figure 10:**
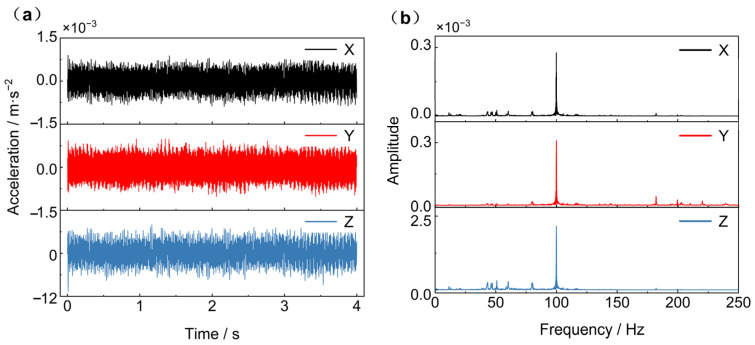
Micro-vibration source data (**a**) Acceleration time domain signal data; (**b**) Acceleration frequency domain signal data.

6 accelerometers were uniformly placed on the infrared refrigerator mounting frame to measure the micro-vibration signals. A data point was collected every 0.002 s for a continuous measurement of 4 s for a total of 2000 moments. The acceleration signals measured by six accelerometers were averaged to obtain micro-vibration signals. The curves in time domain and frequency domain were shown in Figure 10. The amplitude of acceleration in the Z-direction is approximately 10 times that in the X and Y directions. Through Fourier transform of the time-domain data, the frequency domain range of 0–250 Hz is obtained. Characteristic peaks are observed at 43.3 Hz, 46.0 Hz, 50.8 Hz, 60.3 Hz, 80.2 Hz, and 99.8 Hz in all three directions. Among these, the amplitude at 99.8 Hz is the largest.

### 4.2. Opto-Mechanical Integration Analysis Process

Firstly, the optical system model is established through Zemax OpticStudio Standard Edition software, and the corresponding mechanical structure model is established through NX according to the design parameters of the optical model. SiC is used for the optics in the mechanism and lightweight aluminium alloys are used for the mounts and bases. The flexible joints and gaskets are made of indium steel, and the connecting rods between the primary mirror and the secondary mirror are made of carbon fibre, which has a very low coefficient of thermal expansion. The rest of the structure is made of titanium alloy, taking into account the need for strength, stiffness and lightness.

**Figure 11:**
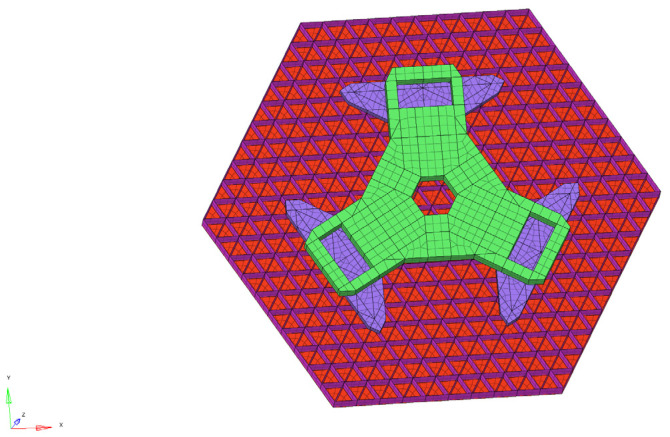
Shell cell model of a sub-mirror assembly.

In the modeling process, the thin-walled lightweight parts can be well simulated by using shell units to model the reflector assembly. Finite element modeling is widely used in engineering design and is a proven means of engineering analysis with high accuracy in dealing with linear models. In the selection of the reflector cell size, no less than three layers of mesh are designed based on experience, as shown in Figure 11.

**Figure 12:**
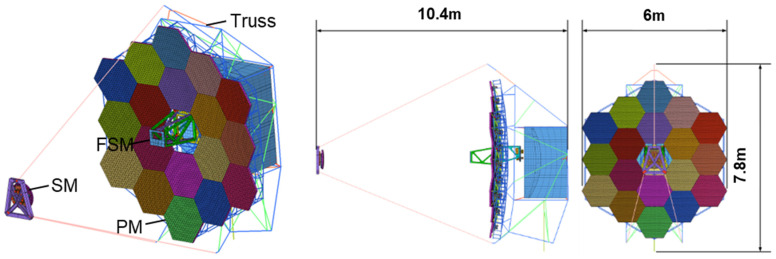
Finite element modeling of segmented telescopes.

The truss is modeled by one-dimensional Beam cells as shown in Figure 12 below. To model the degrees of freedom of the truss rods well, each rod consists of more than 20 cells. The number of nodes in the finite element model of the whole machine is 251,765 and the number of cells is 278,139. Considering the special working conditions of the on-board satellite operation in orbit, the model adopts the free-freedom boundary conditions without any displacement constraints, and the first six-order modes of the model are shown in Table 2.

In terms of the verification of finite element simulation accuracy, there are generally two ways to verify. On the one hand, one can judge whether the results converge by continuously reducing the cell size and increasing the degrees of freedom of the model. On the other hand, the accuracy of finite element simulation can be verified by physical objects. The present finite element model is verified to satisfy the accuracy requirements by the first method.

The micro-vibration source is loaded on the support structure of the optical system, and the coordinate values of each node on the primary mirror surface before and after the change of 2000 moments are obtained by transient analysis. The error equation is constructed by the coordinate transformation relation, and the rigid body displacement of each sub-mirror is calculated by the least square method. Because the influence of the sub-mirror shape change on the WFE is negligible compared with that of the rigid body displacement, the surface shape change of the sub-mirror is not considered.

The 2000 time points of the micro-vibration source signal correspond to 2000 states of the sub-mirrors, and the 18 sub-mirrors have 6 degrees of freedom change, the change of the data volume is large, so the real-time data transmission and read the simulation results of the data interface is written. Using the built-in DDE (Dynamic Data Exchange) server of the optical design software, a dynamic link is established with the numerical computation software, and the changes in the degrees of freedom of the sub-mirrors at each moment are transmitted to the optical design software for analysis, so that the evaluation parameters of the optical system at each moment can be obtained.

The 18 sub-mirrors of the segmented primary mirror have 6 degrees of freedom of change at each moment, and the amount of change data is large. It is very inefficient to change the optical structure data of each moment manually and easy to input errors. Therefore, use MATLAB to write a real-time transmission of data and read the simulation results of the data interface. ZEMAX built-in Dynamic Data Exchange(DDE) server is used to establish a dynamic link with MATLAB. Each moment of the sub-mirror degrees of freedom change amount transferred to ZEMAX and analysis, get the evaluation parameters of the optical system at each moment.

### 4.3. Imaging Quality of Optical Systems Under Micro-Vibration

The results of opto-mechanical integration analysis under micro-vibration show that the WFE RMS is satisfied to be less than 0.061λ for the vast majority of the moments in 4 s, and the RMS is satisfied to be less than 0.071λ for the whole time period.

**Figure 13:**
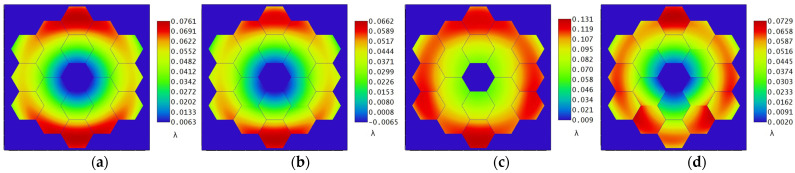

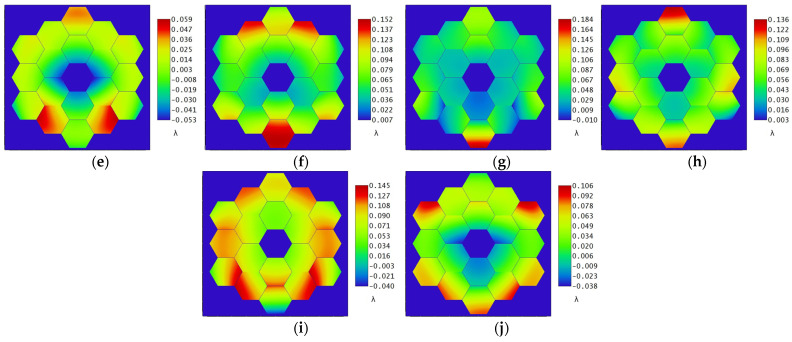
10 consecutive moments wavefront chart (**a**) t = 0 s; (**b**) t = 0.002 s; (**c**) t = 0.004 s; (**d**) t = 0.006 s; (**e**) t = 0.008 s; (**f**) t = 0.010 s; (**g**) t = 0.012 s; (**h**) t = 0.014 s; (**i**) t = 0.016 s; (**j**) t = 0.018 s.

The wavefront diagrams of 10 consecutive moments are taken as shown in Figure 13, where (a) shows the initial system WFE at 0 s. The scale on the right of the figure can directly see the PV value of the WFE at this moment. The micro-vibration source input signal of micro-vibration has randomness in the time domain, and the micro-vibration signal is transmitted to the primary mirror of the optical system causing the position change of the sub-mirror to have a certain degree of randomness. By establishing the sensitivity matrix of the relationship between the WFE and the position of the primary mirror, the positional attitude of the primary mirror can be deduced from the detected wavefront, which provides ideas for the mounting of the primary mirror of the segmented mirror.

When the optical system is under long exposure time, its actual imaging effect is the energy accumulation under the uniform time interval, and the PSF of the optical system under long exposure time is the superposition of the PSF obtained at each moment in the integration time. Under the action of micro-vibration, the shift of the system’s optic axis will lead to the shift of the center position of the PSF. Secondly, due to the change of the position between the sub-mirrors caused by the change in the shape of the primary mirror, so that the energy distribution of the PSF is also changed accordingly, and the joint effect of the two will make the system PSF fuzzy.

**Figure 14:**
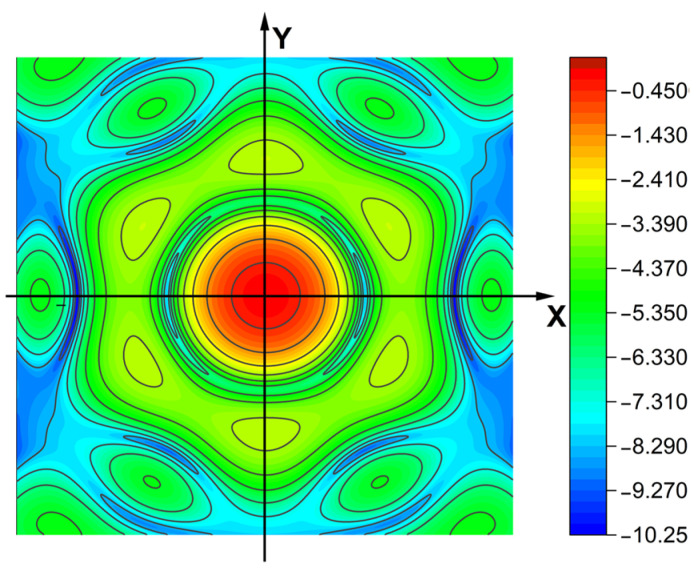
PSF distribution at the initial moment (taking log values).

As shown in Figure 14, the PSF of the center field of view under the initial moment of the optical system (taking the log value), due to the symmetric arrangement of the primary mirror’s sub-mirrors, the PSF of the system also has a symmetric distribution about the X and Y axis. At this moment, the PSF has obvious main peaks and side flaps, and the impact on the imaging quality of the optical system is small due to the small magnitude of the micro-vibration amplitude.

**Figure 15:**
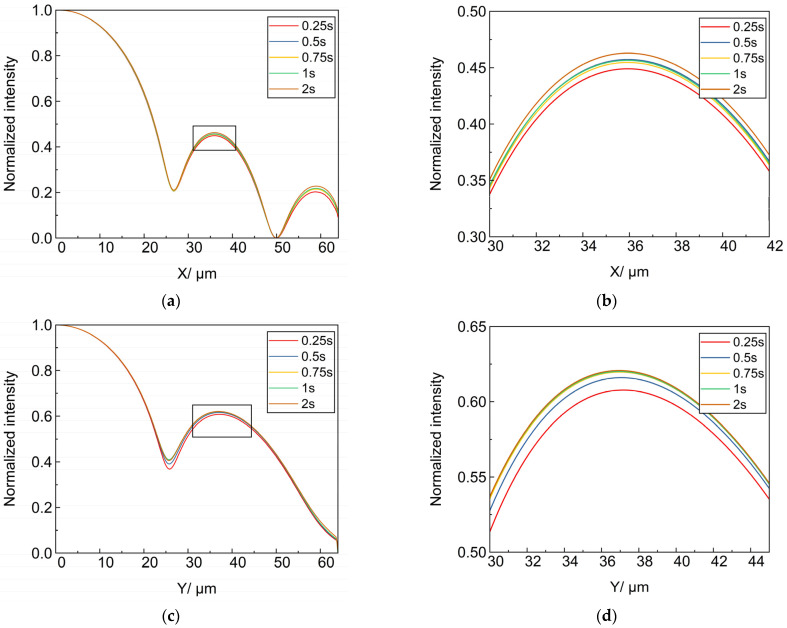
X- and Y-axis cross-sections of PSF at different exposure times (**a**) X-axis cross-section; (**b**) Localized magnification of the secondary peak of X-axis cross-section; (**c**) Y-axis cross-section; (**d**) Localized enlargement of the secondary peak of Y-axis cross-section.

In order to study the influence of micro-vibration on the imaging quality characteristics under different exposure times, the X and Y-axis cross-sections of PSF images with exposure times of 0.25 s, 0.5 s, 0.75 s, 1 s and 2 s were taken respectively, and the actual imaging effect under the long exposure time is the accumulation of energy under the uniform time interval, so the longer the exposure time is, the greater the PSF intensity is. The above exposure time starts from the initial moment of the Micro-vibration source signal in Figure 10, and the PSF with center intensity shifts are accumulated for each moment. The PSF intensity under different exposure times is normalized by taking the positive direction of X and Y axes as shown in Figure 15, respectively (taking the log value), in which (a) is the X-axis cross-section, (b) is the local magnification at the sub-peak of the X-axis cross-section, (c) is the Y-axis cross-section, and (d) is the local magnification at the sub-peak of the Y-axis cross-section.

Under the action of micro-vibration, the secondary peak and the third peak of PSF with different exposure times are gradually separated. The spatial distribution of PSF is related to the resolution ability of the optical system. The higher the resolution, the sharper the main peak of PSF and the smaller the secondary peak, which indicates that the optical system is more effective in imaging. Take the secondary peak value and the main peak value to do the ratio, and get the ratio results as shown in Table 3.

From the table, it can be concluded that for the section along the X-axis, the ratio of the secondary peak to the main peak value is 0.4490 at an exposure time of 0.25 s, and 0.4628 at an exposure time of 2 s. For the section along the Y-axis, the ratio is 0.6077 at an exposure time of 0.25 s, and 0.6207 at an exposure time of 2 s. For both X-axis and Y-axis, the longer the exposure time, the greater the ratio of secondary peak to main peak, this result indicates that the more severe the effect of micro-vibration on image quality.

## 5. Discussion

The effects of translation and tilt of the primary mirror on the WFE of the segmented primary mirror are comprehensively analyzed by theory and simulation. Piston error and Tip & Tilt error have the most significant effect on wavefront error. The results show that, for the decenter of the X-axis (Y-axis) direction of the sub-mirror, the farther away from the coordinate axis the position of the sub-mirror is, the more obvious the influence of the WFE is. The Piston error in the Z direction is more serious than the decenter, and the RMS of the wavefront of the system is increased to 0.45λ when the Piston error is 2 μm. The WFE caused by the tilting of the sub-mirror is not obviously related to the position of the sub-mirror in the range of 1 μrad, and when the tilting of the sub-mirror is 1 μrad, the RMS of the system is 0.15λ. The sub-mirror Clock error has a smaller effect on the wavefront, and the error tolerance is 10^3^ times larger than that of the tip & tilt error.

The opto-mechanical integration analysis of the micro-vibration of the optical system is carried out by using the established dynamic data connection. With an acceleration amplitude of 10 ^−3^m/s^−2^, the system’s WFE RMS over a 4 s time horizon was better than 1/14λ at most moments. The shape of PSF is blurred and the center of PSF is shifted by micro-vibration. The ratio of secondary peak to main peak at different exposure time is used as the evaluation parameter. When the exposure time is 2 s, the ratio of X-axis to Y-axis reaches the maximum of 0.4628 and 0.6207, and the imaging effect is the worst at this time. The simulation process can do micro-vibration simulation test on the ground for segmented telescope, which can provide ideas for the optimization of micro-vibration isolation structure and active optical control.

## 6. Conclusions

A simulation method is proposed for a segmented telescope with a single sub-mirror in the presence of position errors, and the influence of different kinds of position errors on the WFE of the optical system is analyzed and compared. The results of this analysis show the positional errors that should be focused on during the mounting and active control stages of the segmented telescope. Meanwhile, an optical-mechanical integration simulation method for establishing dynamic links is used to obtain the optical imaging quality of the segmented telescope under micro-vibration, which makes the study more accurate, efficient or reliable. A method of evaluating the image quality of the optical system under micro-vibration at exposure time is also proposed, which adopts the peak ratio as the evaluation index and can effectively reflect the imaging effect.

In future work, the positional states of PSF with different shape distributions of the optical system corresponding to different sub-mirrors will be investigated and links will be established to provide ideas for subsequent states based on segmented telescopes.

## Figures and Tables

**Table 1:** Optical system parameter.

Surface	Radius/mm	Thickness/mm	Conic
PM	−15,879.70	−7169.00	−0.99670
SM	−1778.90	7965.30	−1.65980
TM	−3016.20	−1844.10	−0.65950
FSM	+∞	3027.61	0
Image	−3040.46	-	-

**Table 2:** First 6 orders of modal shapes.

Modal	Characteristic Frequency/Hz	Vibration Pattern
1	7.56	X-direction vibration of the secondary mirror
2	9.50	Y-direction vibration of the secondary mirror
3	128	Secondary mirror Z-axis rotation
4	174	Z-direction vibration of the whole machine
5	191	Y-direction vibration of the secondary mirror
6	194	X-direction vibration of the secondary mirror

**Table 3:** Ratio of PSF sub-peak to main peak value.

Exposure Time/s	X-Axis Ratio	Y-Axis Ratio
0.25	0.4490	0.6077
0.5	0.4572	0.6160
0.75	0.4546	0.6197
1	0.4565	0.6201
2	0.4628	0.6207

## Data Availability

The data presented in this study are available on request from the corresponding author.
